# BMI-for-age graphs with severe obesity percentile curves: tools for plotting cross-sectional and longitudinal youth BMI data

**DOI:** 10.1186/s12887-017-0885-x

**Published:** 2017-05-24

**Authors:** Susan B. Racette, Liyang Yu, Nicholas C. DuPont, B. Ruth Clark

**Affiliations:** 1 Washington University School of Medicine, Campus Box 8502 4444 Forest Park Avenue, St. Louis, MO 63108 USA; 2 Washington University School of Medicine, Campus Box 8067, 660 S. Euclid Avenue, St. Louis, MO 63110 USA

**Keywords:** Body mass index, Obesity, Overweight, Excessive body weight, Children, Adolescents, Graphing tool, Weight status

## Abstract

**Background:**

Severe obesity is an important and distinct weight status classification that is associated with disease risk and is increasing in prevalence among youth. The ability to graphically present population weight status data, ranging from underweight through severe obesity class 3, is novel and applicable to epidemiologic research, intervention studies, case reports, and clinical care.

**Methods:**

The aim was to create body mass index (BMI) graphing tools to generate sex-specific BMI-for-age graphs that include severe obesity percentile curves. We used the Centers for Disease Control and Prevention youth reference data sets and weight status criteria to generate the percentile curves. The statistical software environments SAS and R were used to create two different graphing options.

**Results:**

This article provides graphing tools for creating sex-specific BMI-for-age graphs for males and females ages 2 to <20 years. The novel aspects of these graphing tools are an expanded BMI range to accommodate BMI values ˃35 kg/m^2^, inclusion of percentile curves for severe obesity classes 2 and 3, the ability to plot individual data for thousands of children and adolescents on a single graph, and the ability to generate cross-sectional and longitudinal graphs.

**Conclusions:**

These new BMI graphing tools will enable investigators, public health professionals, and clinicians to view and present youth weight status data in novel and meaningful ways.

**Electronic supplementary material:**

The online version of this article (doi:10.1186/s12887-017-0885-x) contains supplementary material, which is available to authorized users.

## Background

The American Heart Association’s Scientific Statement *Severe Obesity in Children and Adolescents: Identification, Associated Health Risks, and Treatment Approaches* [1] highlights the significance of severe obesity among youth in the U.S. and establishes a standard definition of severe obesity for children and adolescents. The most recent report on obesity among children and adolescents [2] indicates that 17.0% of youth aged 2 to 19 years were categorized as obese in 2011–2014; 5.8% of the sample was further classified as having severe (also referred to as extreme) obesity. These prevalence estimates are based on a sample of 6878 youth whose height and weight were measured as part of the National Health and Nutrition Examination Surveys.

The serious health consequences of severe obesity [1, 3] necessitate attention to this problem, with efforts toward screening, treatment, and prevention. Screening initiatives have been conducted in many large urban school districts [4–6] and other populations throughout the U.S. [7] to identify youth who are at greatest risk for adverse health outcomes associated with severe obesity. Intervention approaches in communities, schools, and clinical settings have varying degrees of effectiveness, but are essential to explore for their potential benefits for individuals and for public health. The American Heart Association’s 2016 Scientific Statement *Cardiovascular Health Promotion in Children: Challenges and Opportunities for 2020 and Beyond* [8] emphasizes the importance of improving cardiovascular health metric scores among children with obesity, as obesity is one of seven characteristics that defines poor cardiovascular health in children and adolescents. Due to the challenges of long-term treatment efficacy, however, prevention efforts are essential [9].

All of these approaches – screening, treatment, and prevention – can benefit from the ability to plot youth body mass index (BMI) data on BMI-for-age graphs for visual depiction of weight status and the extent of severe obesity in a clear and informative manner. Tracking the weight status of children and adolescents over time, whether in research-based interventions, epidemiologic studies, or medical treatment programs, is another important application of BMI-for-age graphs. The program Epi-Info [10], available for free download from the CDC website, enables graphing a single child’s BMI over time. This powerful program has extensive capabilities, but currently does not include severe obesity percentile curves and does not enable data from more than one child to be plotted on a single graph. Automated methods for plotting BMI data of multiple youth on sex-specific BMI-for-age graphs that include severe obesity percentile curves are needed and will be valuable for public health and research initiatives.

The Centers for Disease Control and Prevention (CDC) has a publicly available BMI SAS program [11] to compute sex- and age-specific BMI percentiles and BMI z-scores for the determination of weight status of children and adolescents. Our goal was to build upon existing resources and provide tools for investigators, public health professionals, and clinicians to plot youth BMI data on BMI-for-age graphs containing severe obesity percentile curves.

## Methods

The aim of these graphing tools is to facilitate presentation of individual-level BMI data of large groups of children and adolescents, including those with excessive body weight (i.e., severe obesity). We present graphing tools to generate cross-sectional or longitudinal BMI-for-age graphs in an automated manner using the statistical software environments SAS and R.

### Weight status classification

We categorized weight status according to the 2007 Expert Committee recommendations [12], with the additional category of severe obesity described by Flegal et al. in 2009 [13] and defined in a Scientific Statement of the American Heart Association in 2013 [1]. In addition, we included two distinct classes of severe obesity defined by Skinner and Skelton in 2014 [14]. The resulting six weight classifications are underweight (BMI-for-age <5th percentile), healthy weight (5th to <85th percentile), overweight (85th to <95th percentile), obese class 1 (95th percentile to <120% of the 95th percentile), severe obesity class 2 (120% to <140% of the 95th percentile or BMI 35.0 to <40.0 kg/m^2^), and severe obesity class 3 (BMI ≥ 140% of the 95th percentile or BMI ≥ 40.0 kg/m^2^).

### Reference data sets

The reference population used to determine BMI percentiles and z-scores is based on a large sample of children and adolescents whose height and weight were measured as part of the National Health Examination Surveys (NHES) and the National Health and Nutrition Examination Surveys (NHANES) conducted between 1964 and 1994. NHES and NHANES are part of the National Center for Health Statistics (NCHS) data sets that were used to develop the 2000 CDC growth charts [15]. The NCHS reference data set needed to produce the results output was obtained from the CDC website [11] in two file formats: SAS bat file (cdc_ref.sas7bdat) and an Excel csv file (cdcref_d.csv); both are provided as Additional files with this article. The NCHS reference data used to produce the percentile curves on the BMI-for-age graphs were obtained from tables on the CDC website [16] and are provided as an Additional Excel file (Ref_percentile_curves.xlsx).

### Preparing youth data for graphing

Table 1 lists the data inputs required to use the graphing tools. For cross-sectional data sets, each participant ID number must be unique. When participant ID numbers are replicated within a data set, the graphing programs treat the data as longitudinal. Sex is an essential variable because BMI percentiles and z-scores are sex-specific. Age should be computed from date of birth and date of assessment and expressed to one or more decimal places for greatest accuracy. Self-reported age as a whole number may lead to misclassification of weight status, particularly for young children. Height (cm) and weight (kg) must be provided in metric units; height and weight data obtained in English units should be converted to metric units: height (cm) = height (inches) * 2.54; weight (kg) = weight (lbs) / 2.20462. If height and weight data are not available but BMI data are available, then BMI (kg/m^2^) can be provided instead. The graphing programs utilize height and weight data preferentially to compute BMI, BMI percentiles, and BMI z-scores. In the absence of height and weight values, investigator-provided BMI values will be used to compute BMI percentiles and z-scores. Age, height, weight, and BMI (if provided) should be expressed to the greatest degree of accuracy for which the measurement was obtained; rounding may reduce the accuracy of the computed BMI values.

**Table 1:** Data Inputs Required and Results Output

	File Name	File Format	Variable Name	Description
Data Inputs	BMI_Data	Excel Spreadsheet (.xlsx or.xls)	ID	numeric
			Sex	F, M, female, or male
			Age_y	age in years
			Height_cm	height in cm
			Weight_kg	weight in kg
			*BMI*	*kg/m* ^*2*^ *; needed only if height or weight is not provided*
Results Output	BMI_Results	Excel Comma Separated Values (.csv)	BMI_kgm2	BMI in kg/m^2^
			BMI_pct	BMI percentile
			BMI_z	BMI z-score
			BMI_95	BMI as a percent of the 95th percentile
			Weight_status	underweight, healthy weight, overweight, obese class 1, severe obesity class 2, severe obesity class 3

Legend: Data inputs reflect variable names and formats required in the investigator’s data file. Results output represent the variables generated by the SAS and R graphing programs.

### Graphing programs and files needed

Graphing program files are provided for the statistical analysis software SAS (SAS Institute Inc., Cary, NC) and the statistical computing and graphing environment R [17], as described below. The results output files generated by SAS and R contain identical results; the graphs generated by SAS and R display the same data points and curves and are similar in appearance.

SAS: Investigators who choose to use SAS must have SAS software and the six files listed in Table 2 to generate the results file and BMI-for-age graphs. The first file is the SAS graphing program, two files are SAS macro files, two files are CDC reference data sets, and the sixth file is the investigator’s data set. The first five files are provided as Additional files; these files must be accessible on the investigator’s computer or network and should be placed in the same folder as the investigator’s data file. SAS version 9.4 was used to create the SAS graphing program.

**Table 2:** Files Required for Generating Graphs Using SAS and R

	File Name	Description
SAS	Additional file 1	SAS graphing program file
	Additional file 2	SAS Macro from the CDC website
	Additional file 3	SAS Global Forum 2010 %DROPMISS Macro
	Additional file 4	CDC reference data set to compute individual percentiles and z-scores
	Additional file 5	CDC reference data set to generate the percentile curves on the BMI-for-age graphs
	BMI_Data.xlsx	Investigator’s BMI data file
R	Additional file 6	R graphing program file
	Additional file 7	CDC reference data set to compute individual percentiles and z-scores
	Additional file 5	CDC reference data set to generate the percentile curves on the BMI-for-age graphs
	BMI_Data.xlsx	Investigator’s BMI data file

Legend: All files listed in this table, except the Investigator’s Data file, are provided as Additional files with this article.

R: Investigators who choose to use R must have R software and the four files listed in Table 2 to generate the results file and BMI-for-age graphs. The first file is the R graphing program, two files are CDC reference data sets, and the fourth file is the investigator’s data set. The first three files are provided as Additional files; these files must be accessible on the investigator’s computer or network and should be placed in the same folder as the investigator’s data file. We used R version 3.3.0 to create the R graphing program file. R software is available for download free of charge.

## Results

### Data file containing BMI percentile, BMI z-score, and weight status

The results output file is an Excel csv file (BMI_Results.csv) that contains the data input variables provided by the investigator plus the output variables indicated in Table 1. BMI (kg/m^2^) is computed as weight (in kg) divided by height (in meters) squared. Sex- and age-specific BMI percentiles and BMI z-scores are determined based on the reference population. BMI as a percent of the 95th percentile (BMI_95) is an important metric for identifying severe obesity. Weight status is determined based on BMI percentile and BMI as a percent of the 95th percentile. Each weight status category is mutually exclusive, with obesity classes 1, 2, and 3 being distinct and all weight status categories summing to 100%. Therefore, if investigators wish to determine the prevalence of obesity in their sample, they must add the three classes of obesity. Likewise, to determine the prevalence of severe obesity, it is necessary to add obesity classes 2 and 3.

### BMI-for-age graphs

**Fig. 1:**
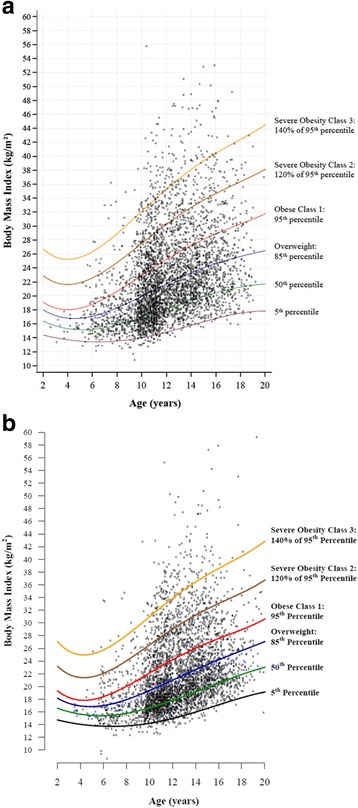
BMI-for-age graphs showing cross-sectional BMI data for 3900 females (**a**) and 4000 males (**b**). Graph A was generated using SAS; graph B was generated using R. Each symbol represents the BMI value of a single child or adolescent. Data were drawn from published [6, 19, 20] and unpublished studies

**Fig. 2:**
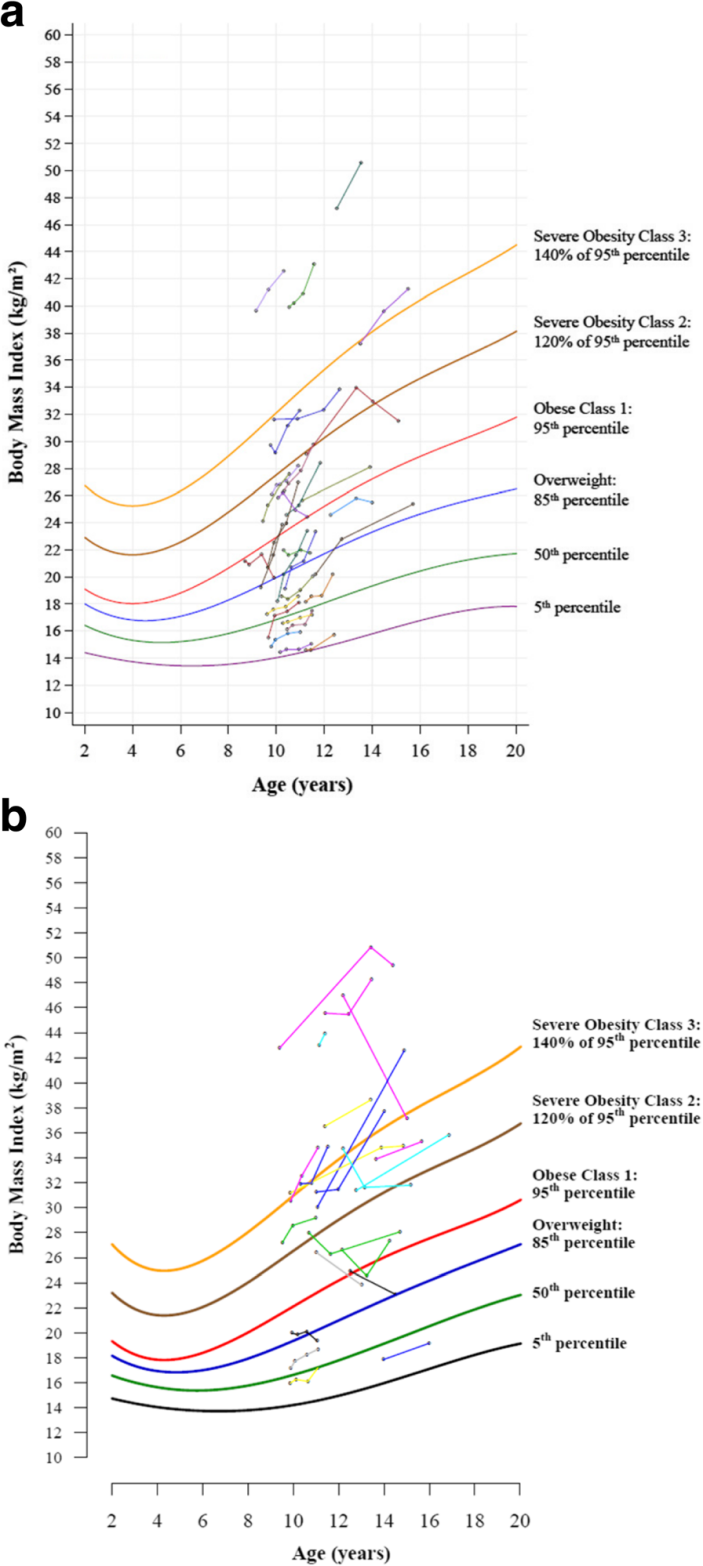
BMI-for-age graphs showing longitudinal BMI data for 30 females (**a**) and 22 males (**b**). Graph A was generated using SAS; graph B was generated using R. Each circle represents one measurement; each set of circles connected with a line represents one child. Data were drawn from published [6, 19, 20] and unpublished studies

Table 3 lists the file names and file formats for the BMI-for-age graphs generated by SAS and R. These graphs can be output as Adobe Illustrator Encapsulated PostScript (.eps) vector graphics files and/or as Adobe Acrobat Portable Document Format (.pdf) files. Figs. 1 and 2 are provided as examples of cross-sectional and longitudinal graphs generated using the SAS and R program files. These graphs are provided for illustrative purposes only; the data contained in them were drawn from a series of published [6, 18, 19] and unpublished studies that were approved by the Washington University in St. Louis Institutional Review Board.

**Table 3:** BMI-for-Age Graphs Generated Using SAS and R

	File Name	File Format	Data Type
SAS	BMI_Graph_females_sas	.eps or. pdf	Cross-sectional
	BMI_Graph_males_sas	.eps or. pdf	Cross-sectional
	BMI_Graph_females_long_sas	.eps or. pdf	Longitudinal
	BMI_Graph_males_long_sas	.eps or. pdf	Longitudinal
R	BMI_Graph_females_R	.eps or. pdf	Cross-sectional
	BMI_Graph_males_R	.eps or. pdf	Cross-sectional
	BMI_Graph_females_long_R	.eps or. pdf	Longitudinal
	BMI_Graph_males_long_R	.eps or. pdf	Longitudinal

Our BMI-for-age graphs are designed to mimic the CDC’s 2000 sex-specific BMI-for-age growth charts for males and females [15, 20], with age on the x-axis, BMI (kg/m^2^) on the y-axis, and several standard percentile curves (i.e., 5th, 50th, 85th, and 95th) displayed on each graph. Four distinct features of these new BMI-for-age graphs are an expanded BMI range to accommodate youth with severe obesity, the inclusion of the two severe obesity percentile curves (i.e., 120% of the 95th percentile to signify the cut point for obese class 2 and 140% of the 95th percentile as the cut point for obese class 3), the ability to plot multiple children and adolescents on each graph, and the ability to generate cross-sectional and longitudinal graphs.

### Cross-sectional BMI-for-age graphs

Figure 1a and b depict cross-sectional data for 3900 females and 4000 males, respectively, aged 2 to <20 years, whose height and weight were measured. Each symbol represents a single child or adolescent, with his/her age apparent from the x-axis, BMI indicated on the y-axis, and weight status reflected by the position relative to the percentile curves. Points above the top two percentile curves on each graph reflect severe obesity (i.e., obese classes 2 and 3). These scatterplots enable one to view the extent of obesity and severe obesity quickly and easily.

### Longitudinal BMI-for-age graphs

Figure 2a and b depict longitudinal data for 30 females and 22 males whose height and weight were tracked for up to four years. Each set of circles with a connecting line represents one child; each circle represents one measurement time point. The two informative aspects of these graphs are: 1) the BMI-for-age percentile at each measurement time point represents the child’s weight status at that time point and 2) the slope of each child’s line relative to the percentile curves represents an increase, no change, or a decrease in BMI-for-age percentile over time. A steep upward slope, as is evident for some participants, signifies a rapid (and potentially undesirable) increase in BMI.

## Discussion

In this article we describe and provide programs for graphing youth BMI data on sex-specific BMI-for-age graphs that contain the four traditional weight categories (underweight, healthy weight, overweight, obese) plus two categories of severe obesity (obese classes 2 and 3). The graphing tools provided here utilize the National Center for Health Statistics reference data on children and adolescents and the CDC’s BMI SAS code, expanding the capabilities of those valuable resources to facilitate graphical presentation of cross-sectional and longitudinal youth BMI data for investigators, public health professionals, and clinicians.

A currently available resource to generate a BMI-for-age graphs for an individual youth is the widely-used program Epi-Info [10]. Three additional features of our graphing tools that are not part of Epi-Info are inclusion of severe obesity percentile curves, capacity for thousands of youth on a single graph, and the option to generate cross-sectional or longitudinal graphs. The CDC has publicly available Excel files containing macros to compute age- and sex-specific BMI percentiles and to generate summary bar graphs depicting the prevalence of overweight and obesity for a group of up to 2000 children [21], which is a valuable resource for schools. In comparison with our new graphing tools, the CDC macro does not have the capacity to generate BMI-for-age graphs and does not distinguish severe obesity.

### Modifications to accommodate investigators’ data sets and preferences

Investigators and other individuals who use these graphing tools may modify the SAS and R program files to accommodate the BMI range of their data (i.e., setting the y-axis range lower or higher than 60 kg/m^2^), the input data file format (e.g., .xlsx, .csv, .bat), and graph preferences (e.g., symbol style, size, and color; line color and thickness; and font style and size). Also, investigators can insert additional code into the beginning of the SAS and R program files to compute age (from date of birth and date of assessment) and to convert height and weight from English to metric units. Other modifications that may be desirable include inserting additional percentile curves on the BMI-for-age graphs (e.g., 3rd, 10th, 25th 75th, 90th, 97th, 99th).

### Strengths and limitations

Strengths of these graphing tools are their novelty, the inclusion of severe obesity percentile curves, the ability to plot thousands of youth on each graph, and the flexibility to plot cross-sectional or longitudinal data. A limitation is the need for either SAS or R software and reference data sets.

## Conclusions

An alarming number of children and adolescents have severe obesity, which has significant health consequences. The BMI-for-age graphing tools presented in this article facilitate graphical presentation of cross-sectional and longitudinal youth weight status data ranging from underweight through severe obesity class 3 for use in epidemiologic research, intervention studies, case reports, and clinical care. These new graphing tools will enable investigators, public health professionals, and clinicians to view and present individual-level BMI data in novel and meaningful ways.

## Additional files

Additional file 1: BMI SAS Graphing Program.sas. SAS graphing program file (SAS 11 kb)

Additional file 2: cdc-source-code.sas. SAS Macro from the CDC website (SAS 7 kb)

Additional file 3: DROPMISS_MACRO.sas. SAS Global Forum 2010 %DROPMISS Macro (SAS 5 kb)

Additional file 4: cdcref_d.sas7bdat. CDC reference data set to compute individual percentiles and z-scores (SAS7BDAT 305 kb)

Additional file 5: Ref_percentile_curves.xlsx. CDC reference data set to generate the percentile curves on the BMI-for-age graphs (XLSX 69 kb)

Additional file 6: BMI R Graphing Program.R. R graphing program file (R 10 kb)

Additional file 7: cdcref_d.csv. CDC reference data set to compute individual percentiles and z-scores (CSV 159 kb)

## Abbreviations

BMI: Body Mass Index
CDC: Centers for Disease Control and Prevention
